# Cytomegalovirus Infection Mimics Manifestations of Underlying Diseases in Patients With Autoimmune Disorders: A Case Report and Literature Review

**DOI:** 10.1002/iid3.70377

**Published:** 2026-03-18

**Authors:** Elaheh Karimi, Zahra Moradi, Somayeh Soroureddin, Ozra Nouri, Zahra Tamartash, Hoda Kavosi

**Affiliations:** ^1^ School of Medicine Tehran University of Medical Sciences Tehran Iran; ^2^ Rheumatology Research Center Tehran University of Medical Sciences Tehran Iran

**Keywords:** AMAN syndrome, autoimmune diseases, cutaneous ulcer, cytomegalovirus

## Abstract

**Background:**

Patients with autoimmune disorders are highly susceptible to infections including cytomegalovirus (CMV) leading to serious complications ranging from asymptomatic to severe systemic diseases.

**Case Presentation:**

The first case was a 44‐year‐old woman with systemic lupus erythematosus (SLE) referred to the Rheumatology ward due to a necrotizing ulcer on the hand's finger and multiple ulcerative lesions on her lips and tongue. She had pancytopenia and tested positive for both herpes simplex virus (HSV) and CMV polymerase chain reaction (CMV‐PCR). She was treated with ganciclovir for 14 days and subsequently recovered. The second case was a 55‐year‐old man, a known sarcoidosis case, admitted to the hospital due to lower extremities weakness and intraoral ulcers. The lab findings revealed leukopenia and elevated levels of ESR and CRP. Viral markers were all negative except for the CMV‐PCR test. Electromyography and nerve conduction velocity (EMG‐NCV) showed subacute axonal motor polyneuropathy in the lower limbs. Due to the high titers of CMV (500,000 copies/mL), he was treated with ganciclovir for 2 weeks and the symptoms improved dramatically.

**Conclusion:**

CMV infection in patients with inflammatory rheumatic diseases may lead to rare manifestations that can be misdiagnosed as a flare of the underlying disease.

## Graphical abstract

Autoimmune disorder patients are vulnerable to cytomegalovirus (CMV) infections, which can present with symptoms that mimic flare‐ups of the underlying disease. We present two cases: a 44‐year‐old woman with systemic lupus erythematosus and a 55‐year‐old man with sarcoidosis, both of whom developed unusual manifestations linked to CMV infection, confirmed by PCR. Treatment with Ganciclovir led to significant improvement, emphasizing the need for careful consideration of CMV in autoimmune patients with atypical symptoms.

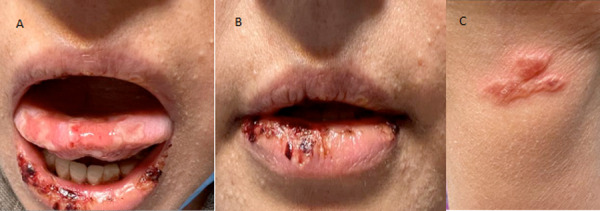

**Abbreviations**
ACE: angiotensin‐converting enzyme
AIDS: acquired immunodeficiency syndrome
AMAN: acute motor axonal neuropathy
CMV: cytomegalovirus
CRP: C‐reactive protein
CSF: cerebrospinal fluid
CT: computed tomography
EMG‐NCV: electromyography and nerve conduction velocity
ESR: erythrocyte sedimentation rate
GBS: Guillain–Barré Syndrome
Hb: hemoglobin
HSV: herpes simplex virus
IV: intravenous
IVIG: intravenous immunoglobulin
LDH: lactate dehydrogenase
PBS: peripheral blood smear
PCR: polymerase chain reaction
SLE: systemic lupus erythematosus
TIBC: total iron‐binding capacity

## 1 Introduction

Rheumatic disorders represent a heterogeneous group of autoimmune and inflammatory diseases affecting multiple organs, most frequently the joints, muscles, bones, and connective tissues [1]. Patients with inflammatory rheumatic diseases (IRDs) are highly susceptible to opportunistic infections, including cytomegalovirus (CMV) infection, which can lead to severe complications [2]. The prevalence of CMV infection in autoimmune diseases is notably high. Recent studies have found that CMV antigenemia is detected in approximately 35% of patients with autoimmune disorders, with prevalence reaching 58% in those with systemic lupus erythematosus (SLE), and 11.4% in individuals with non‐SLE autoimmune diseases [2, 3, 4]. In another study of 834 patients with rheumatic diseases undergoing corticosteroid treatment, 142 (17%) tested positive for CMV DNA [5]. Similarly, sarcoidosis patients are prone to primary CMV infection or reactivation of latent CMV, due to T‐cell dysfunction and corticosteroid therapy [6].

In immunocompromised patients, CMV manifestation may vary from asymptomatic to severe systemic disease involving the gastrointestinal tract, liver, nervous system, cardiovascular system, and lungs [7]. Skin involvement is uncommon, but cases of anogenital ulceration, maculopapular rashes, and cutaneous necrotizing vasculitis have been reported [8, 9, 10].

Neurological manifestations, including Guillain–Barré syndrome (GBS), polyradiculopathy, and sensorineural hearing loss, have also been described in CMV‐infected patients [11, 12, 13].

Distinguishing CMV reactivation from an autoimmune disease flare can be challenging. Given the rarity of CMV manifestations and their difficulty in differentiating them from autoimmune conditions, we present two cases of CMV infection in patients with IRDs with unusual clinical features, highlighting the significance of early detection and accurate differentiation.

## 2 Case 1

### 2.1 Case Presentation

A 44‐year‐old woman was diagnosed with SLE approximately 20 years ago. Her initial symptoms included discoid lesions on the scalp, photosensitivity, Raynaud's phenomenon, oral ulcers, nonscarring alopecia, and positive lupus serology, which confirmed the diagnosis. She had been on long‐term prednisolone (5 mg daily) and hydroxychloroquine (200 mg twice daily) for 20 years, since diagnosis. For the past 4 years, she remained stable without flare‐ups until she developed acute bilateral pitting edema of the legs, dyspnea, hypertension, and elevated creatinine levels. Due to the suspicion of glomerulonephritis, a renal tissue biopsy showed class IV lupus nephritis. Treatment with 1 g of methylprednisolone for three consecutive days, followed by 1 g of cyclophosphamide, improved clinical symptoms and laboratory findings.

One month later, she returned with progressive shortness of breath, productive cough, fever, and chills. Given her long‐term immunosuppressive therapy and recent high‐dose corticosteroid use for lupus nephritis, she was at increased risk for opportunistic infections. Although her initial viral workup, including CMV testing, was negative, delayed viral reactivation due to persistent immunosuppression was considered. She was ultimately diagnosed with pneumonia based on chest computed tomography (CT) findings and laboratory results and was treated with intravenous antibiotics for 14 days. Her symptoms resolved, and she was discharged. However, 2 weeks post‐discharge, she developed a necrotizing ulcer on her left hand and multiple ulcerative lesions on her lips and tongue, prompting her readmission for further evaluation.

### 2.2 Investigations and Treatment

**Figure 1:**
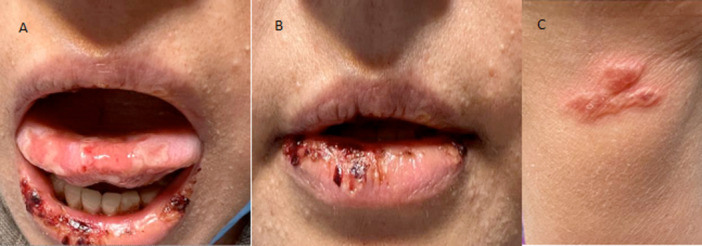
Multiple necrotic ulcers on the tongue (A), lips (B), and the hand (C).

On examination, the patient was afebrile and hemodynamically stable. A full physical examination revealed oral mucocutaneous ulcers, paronychia, ischemic changes, and necrotizing ulcers on the left hand (Figure 1). Laboratory findings showed a hemoglobin level of 8.5 g/dL, a leukocyte count of 4.7 × 10^3^/μL, a platelet count of 92 × 10^3^/μL, an erythrocyte sedimentation rate (ESR) of 82 mm/h, lactate dehydrogenase (LDH) of 980 U/L, and a creatinine level of 4.7 mg/dL. To investigate the cause of anemia, additional tests were performed. Iron deficiency and other etiologies of anemia were excluded, as ferritin, serum iron, total iron‐binding capacity (TIBC), vitamin B12, folic acid, and reticulocyte count were all within normal ranges. Peripheral blood smear (PBS) showed no abnormal cells, ruling out hemolysis or hematologic malignancies.

Given her history of lupus nephritis, rising creatinine levels, and recent discontinuation of cyclophosphamide due to pneumonia, an SLE flare‐up was initially suspected, and prednisolone (1 mg/kg) was started. However, urinalysis revealed decreased proteinuria (from 4+ in previous admission to 1+), and lupus serology, including anti‐double‐stranded DNA (dsDNA) and complement levels (C3 and C4), remained normal. The painful nature of her ulcers and the presence of pancytopenia raised suspicion of viral reactivation rather than an autoimmune flare, prompting further virological testing.

PCR testing for CMV and HSV was conducted to assess viral reactivation. CMV‐PCR on blood samples revealed a viral load of 62,464 copies/mL, confirming significant viremia. HSV‐PCR was also performed on blood, and the qualitative results were positive for HSV. The distinction between CMV and HSV as the primary cause of symptoms was an important consideration in this case. While HSV commonly causes painful oral ulcerations, isolated HSV infection rarely leads to systemic manifestations such as pancytopenia or renal dysfunction, which were present in this patient [14]. In contrast, CMV is known to cause mucocutaneous ulcerations in immunocompromised patients and can also contribute to hematologic abnormalities and worsening renal function [7]. Given the high CMV viral load and the presence of HSV, a mixed herpesvirus infection was the most likely explanation for her clinical symptoms. This case highlights the importance of considering viral infections as potential mimics of autoimmune flares in immunosuppressed patients.

### 2.3 Outcome and Follow‐Up

The patient was treated with intravenous (IV) ganciclovir with dosage adjustments for renal impairment (2.5 mg/kg daily for 14 days). To prevent an SLE flare, intravenous immunoglobulin (IVIG) at 2 g/kg was administered over 5 days. Prednisolone (50 mg daily) was continued during ganciclovir therapy and was later tapered. Hydroxychloroquine (200 mg twice daily) was maintained throughout treatment. Cyclophosphamide was not initiated during the acute viral infection to avoid further immunosuppression.

After completing 14 days of IV ganciclovir, she was transitioned to oral valganciclovir (450 mg daily) for another 14 days. Her necrotizing ulcers and oral lesions gradually improved, and she was discharged. Four weeks post‐discharge, as her CMV viral load had significantly decreased, cyclophosphamide was restarted.

## 3 Case 2

### 3.1 Case Presentation

A 51‐year‐old man with a 6‐year history of sarcoidosis was admitted due to worsening general condition and progressive lower extremity weakness. He had been diagnosed with sarcoidosis in 2019 when he developed progressive shortness of breath and cough. A chest CT scan revealed bilateral hilar lymphadenopathy, diffuse centrilobular nodular opacities, fissural thickening, and alveolar ground‐glass opacities. The diagnosis was confirmed by an elevated CD4/CD8 ratio (> 4.5) and significantly increased angiotensin‐converting enzyme (ACE) levels. However, he declined treatment at that time.

Three years later, he developed blurred vision and eye redness. Slit‐lamp examination and fluorescein angiography revealed retinal vasculitis and pan‐uveitis, prompting initiation of prednisolone (1 mg/kg) and mycophenolate mofetil (2 g/day). His visual acuity improved following treatment. One month later, he presented with fatigue, progressive lower limb weakness, and painful oral ulcers, prompting further evaluation.

### 3.2 Investigations and Treatment

The patient's vital signs were within normal ranges. On neurological examination, muscle strength in the proximal lower limbs was 2/5, with profound weakness in the distal muscles. There were no sensory deficits, and deep tendon reflexes were reduced bilaterally, with a negative Babinski reflex. Additionally, painful intraoral ulcers were observed on the buccal mucosa.

Laboratory tests revealed a leukocyte count of 0.5 × 10^3^/μL, platelet count of 177 × 10^3^/μL, hemoglobin of 14.2 g/dL, C‐reactive protein (CRP) of 20 mg/L, ESR of 45 mm/h, and LDH of 629 U/L. Given the clinical presentation, GBS was suspected, and plasmapheresis was initiated. However, further investigations were necessary to exclude other potential causes.

A brain CT scan and cerebrospinal fluid (CSF) analysis showed no abnormalities. Viral markers, including HIV‐Ab, HCV‐Ab, HBsAg, and PCR‐COVID, were all negative. Due to his immunosuppressed status, a CMV‐PCR test was performed on blood samples, revealing a high viral load of 500,000 copies/mL.

To further assess his neurological deficits, electromyography and nerve conduction velocity (EMG‐NCV) studies were conducted, which showed subacute axonal motor polyneuropathy in the lower limbs, consistent with a diagnosis of acute motor axonal neuropathy (AMAN), a variant of GBS.

### 3.3 Outcome and Follow‐Up

The patient was treated with IV ganciclovir (2.5 mg/kg daily for 14 days). Following the first 7 days of treatment, his oral ulcers showed significant improvement, with complete resolution by the end of the 2‐week antiviral course. His neurological symptoms also improved gradually, with partial recovery of motor function observed after 4 weeks.

After completing 14 days of IV ganciclovir, he was transitioned to oral valganciclovir (450 mg daily) upon discharge to complete antiviral therapy. His condition continued to improve in subsequent follow‐ups.

We reviewed the current literature on mucocutaneous and neurological manifestations of CMV infection in patients with IRDs. Cutaneous CMV presentations were identified in 17 cases, with multiple ulcers being the most prevalent skin lesion. These ulcerations primarily affected the extremities, particularly the lower limbs, followed by the oral cavity, trunk, and perianal area. Additionally, two reported cases exhibited CMV‐induced neurological symptoms, presenting as motor impairment. Treatment strategies included oral valacyclovir in two cases and intravenous ganciclovir in the remainder. Symptom resolution was achieved in most cases, except for four patients who died because of other complications. A summary of the literature review is provided in Table 1.

**Table 1:** Cutaneous and neurological manifestations of CMV infection in patients with inflammatory rheumatologic diseases: A literature review.

No	Author	Sex/age (years)	Underlying disease	Clinical presentations of CMV	Treatment
**Mucocutaneous manifestations**					
1	Fasanya et al. [15]	F/76	Sjögren's syndrome	Diffuse non‐pruritic macular lesion with scattered vesicles and bullae on lower back and buttock	Valganciclovir (oral)
2	Pinana et al. [16]	F/62	SLE	Multiple, painful, and deep oral ulcerations	Ganciclovir (IV) then valganciclovir (oral)
3	Hayes et al. [17]	F/48	Rheumatoid arthritis	Multiple, painful, and perineal ulcerations	Ganciclovir and foscarnet (IV)
4	Srisuttiyakorn et al. [18]	F/66	Rheumatoid arthritis with anti‐phospholipid syndrome	Multiple, ill‐defined, erythematous purpuric patches and pustules on the forearms and legs	Ganciclovir (IV)
5	Srisuttiyakorn et al. [18]	F/49	SLE	Multiple, well‐defined erythematous crusted papules on the forearms, arms, and abdominal wall and a solitary ill‐defined erythematous papule with shallow ulcer and pustules on the elbow	Ganciclovir (IV)
6	Colsky et al. [19]	F/37	SLE	Multiple, painful skin ulcerations on the arms and back	Ganciclovir and foscarnet (IV)
7	Guo et al. [20]	F/64	Mixed connective tissue disease	Two symmetric, well‐circumscribed, oval, and shallow ulcerations on the bilateral lower medial legs	Ganciclovir (IV)
8	Kanetaka et al. [21]	M/83	Dermatomyositis	Erythematous ulceration with raised borders and excoriation on the neck and upper back	Ganciclovir (IV)
9	Kanetaka et al. [21]	M/73	Dermatomyositis	Multiple, erythematous ulceration in the anogenital area and on the trunk	Ganciclovir (IV)
10	Nolan et al. [22]	M/78	Wegener's granulomatosis	Leg ulceration	Valganciclovir (oral)
11	Hajihashemi et al. [23]	F/20	SLE	Painful skin ulceration on the knee	Ganciclovir (IV) then valganciclovir (oral)
12	Agrawal et al. [24]	F/1 Day	Neonatal lupus erythematosus	Confluent macular erythematous rash and petechiae over the face, extensor surfaces of extremities and trunk	No treatment
13	Bhawan et al. [25]	M/54	Vasculitis	Vesiculobullous lesions over the extremities	—
14	Aries et al. [26]	M/63	Wegener's granulomatosis	Palatal mucosal ulcerations	Ganciclovir (IV)
15	Masuda et al. [27]	F/72	Rheumatoid arthritis	Multiple cutaneous ulcers with fluctuating purpuric erythema on lower extremities	No treatment (Died after few days)
16	Faram et al. [28]	F/18	SLE	Multiple oral ulcers	—
17	Huang et al. [29]	M/65	Wegener's granulomatosis	Intraoral ulceration	Ganciclovir (IV)
**Neurological manifestations**					
18	Pavicic Ivelja et al. [30]	F/57	SLE	General impairment, monoparesis, and temporary cognitive disability	Ganciclovir (IV) then valganciclovir (oral)
19	Yun et al. [31]	M/64	Seronegative rheumatoid arthritis	Motor weakness in both lower extremities (lumbosacral polyradiculitis)	Ganciclovir (IV)

Abbreviations: F, female; IV, intravenous; M, male; SLE, systemic lupus erythematosus.

## 4 Discussion

CMV, a β‐herpesvirus, is a significant opportunistic infection in immunocompromised patients, often presenting with mononucleosis‐like syndrome [32]. In patients with IRDs, CMV infection can exacerbate the underlying condition or even trigger disease onset. Reactivation may involve multiple organs, including the liver, lungs, colon, and esophagus, often mimicking autoimmune disease flares and complicating diagnosis [33].

CMV infection is particularly relevant in SLE, occurring in approximately 10% of cases, often with nonspecific symptoms such as fever and fatigue. Cutaneous CMV manifestations vary widely, ranging from oral ulcerations and localized lesions to generalized eruptions that resemble autoimmune rashes [34, 35]. While CMV‐related skin lesions are most commonly seen in acquired immunodeficiency syndrome (AIDS), extra‐genital ulcers are more frequently observed in patients with IRDs [36]. The pathogenesis of CMV‐associated skin ulcers is thought to involve either direct viral infection of endothelial cells, leading to vascular damage and ulcer formation, or an indirect immune‐mediated mechanism involving autoantibody production [37, 38]. Oral CMV lesions are typically deep, painful ulcers with erythematous borders, distinct from lupus‐related oral ulcers, which are well‐demarcated, red, round, atrophic, or blistered. Skin involvement is a significant indicator of systemic infection, contributing to high mortality rates in immunocompromised patients [39, 40].

Beyond cutaneous involvement, CMV infection can also present with neurological symptoms, including sensorineural hearing loss, immune axonal neuropathies, polyradiculopathy, and multifocal neuropathy [41, 42]. While these complications are well‐documented in AIDS, reports on CMV‐related neurological involvement in IRDs remain limited [31].

In our sarcoidosis case, the patient developed motor nerve dysfunction while sensory nerves remained intact. Sarcoidosis is known to affect the nervous system, involving cranial nerves, meninges, brain parenchyma, spinal cord, pituitary gland, and peripheral nerves [43, 44]. In this case, exacerbation of sarcoidosis may be the underlying cause for the neurological manifestation observed, and it is crucial to distinguish this from other possible explanations. EMG‐NCV confirmed axonal polyneuropathy, while CSF analysis was normal, suggesting AMAN, a GBS variant.

GBS, observed in the second case, is an autoimmune polyradiculopathy often triggered by infections such as campylobacter jejuni, CMV, mycoplasma pneumoniae, and Epstein–Barr virus [45]. It has been suggested that infections lead to an immune response, resulting in cross‐reaction with epitopes on the peripheral nerve and multifocal inflammation in myelin sheaths of the spinal cord and peripheral nerves. Previous studies confirmed that anti‐GM2 is associated with CMV‐related GBS. Activation of the complement system leads to membrane attack complex (MAC) formation, resulting in myelin sheath damage and subsequent invasion by macrophages to clear the debris [11, 46]. The concurrent occurrence of CMV‐related GBS and sarcoidosis has not been previously reported, but potential mechanisms include molecular mimicry, autoimmunity, or direct viral invasion of nerves and muscles [47].

One of the primary challenges in autoimmune cases is differentiating between an autoimmune flare and an infectious process, as both can present with overlapping clinical and laboratory findings. In patients receiving full immunosuppressive therapy, a disease flare is typically expected to respond to treatment, with clinical improvement and stabilization of laboratory parameters, including blood counts. In our first case, despite receiving standard‐dose immunosuppressive therapy and showing a reduction in proteinuria, the patient experienced recurrent leukopenia. Given that the patient had already undergone full immunosuppression and had passed the nadir phase of cyclophosphamide, the probability of an autoimmune relapse at this stage was relatively low. This raised the suspicion of an alternative etiology, particularly an infectious cause such as CMV, prompting further diagnostic evaluation. The SLE patient initially presented with necrotizing ulcers and pancytopenia, raising suspicion of an autoimmune flare due to a history of lupus nephritis. However, normal complement levels, the absence of dsDNA elevation, and a significantly elevated CMV viral load confirmed CMV infection rather than an SLE exacerbation. Symptoms resolved completely after antiviral therapy. Similarly, the sarcoidosis patient's neurological deficits, initially attributed to GBS secondary to sarcoidosis, were ultimately linked to CMV, as confirmed by high CMV viral titers, leukopenia, and the exclusion of other infectious causes. Treatment with ganciclovir led to notable neurological improvement, further supporting the diagnosis.

Since CMV reactivation can mimic autoimmune flares, accurate differentiation is essential. Serological and PCR testing remain key diagnostic tools, with CMV‐specific IgM and IgG serology distinguishing recent infection from past exposure. Viral load quantification aids in identifying active infection, while inflammatory and autoimmune biomarkers such as complement levels (C3, C4), anti‐dsDNA titers, and proteinuria help differentiate CMV infection from autoimmune exacerbations [48, 49].

These cases emphasize the importance of early recognition of CMV infection to prevent misdiagnosis and unnecessary immunosuppression. Clinicians should maintain a high index of suspicion in immunocompromised patients presenting with atypical mucocutaneous or neurological symptoms. Prompt antiviral therapy for CMV can significantly improve patient outcomes, reducing morbidity and mortality.

## 5 Conclusion

Our cases highlight the potential for CMV infection to mimic autoimmune disease flares, creating diagnostic challenges. Maintaining a high level of clinical suspicion for CMV in immunocompromised patients is essential to ensure timely antiviral treatment and prevent disease progression or unwarranted immunosuppression. Future research should prioritize the development of standardized diagnostic protocols to enhance the distinction between CMV infections and autoimmune disease exacerbations.

## Author Contributions

**Elaheh Karimi:** investigation, writing – original draft, writing – review and editing. **Zahra Moradi:** investigation, writing – original draft. **Somayeh Soroureddin:** investigation, visualization. **Ozra Nouri:** writing – original draft. **Zahra Tamartash:** supervision, writing – review and editing. **Hoda Kavosi:** conceptualization, project administration, supervision.

## Funding

The authors received no specific funding for this work.

## Ethics Statement

The study protocol was approved by the ethics committee of Tehran University of Medical Sciences (IR.TUMS.SHARIATI. REC.1402.106). Informed consent was obtained from the patients for participation in the study or the use of the tissue.

## Consent

Written informed consent was obtained from the patients to publish this case report and any accompanying data and images. A copy of the written consent is available for review by the Editor‐in‐Chief of this journal.

## Conflicts of Interest

The authors declare no conflicts of interest.

## Data Availability

The authors have nothing to report.
